# Supplementary material for: Inhibition of Ihh Reverses Temporomandibular Joint Osteoarthritis via a PTH1R Signaling Dependent Mechanism
Source: Int J Mol Sci. 2019 Aug 3;20(15):3797. doi: 10.3390/ijms20153797 (PMC6695690; doi:10.3390/ijms20153797)
Supplement: Supplementary file 1 [file ijms-20-03797-s001.pdf]

Supplementary Figures and Legends

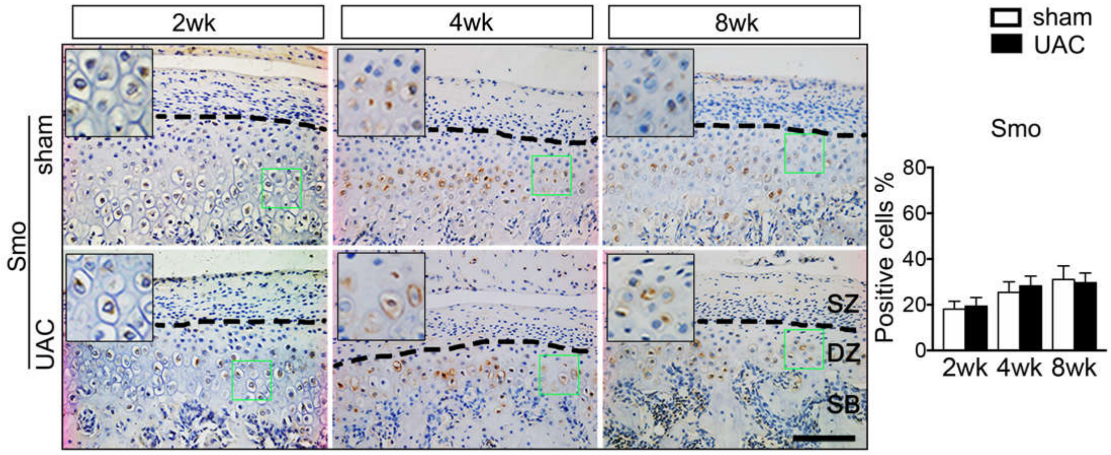

Figure S1. Effects of unilateral anterior cross-bite (UAC) on the expression level of smoothened (Smo) on the sagittal central sections of the TMJ condyles from 2 to 8 weeks. Immunohistochemical staining and quantification of percentage of Smo positive cells. The green box regions in the images were magnified at the top left corner. Dotted line distinguishes the superficial and deep zone chondrocyte. *SZ*, superficial zone. *DZ*, deep zone. *SB*, subchondral bone. Scale bar, 100 m. *n* = 6.

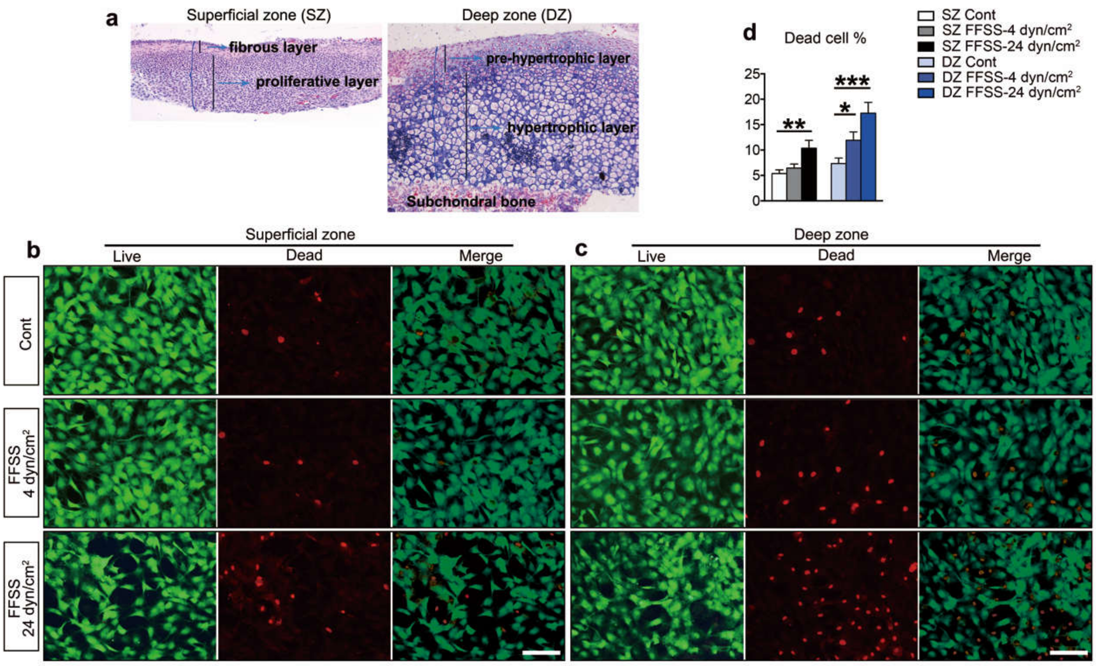

Figure S2. Effects of fluid flow shear stress (FFSS) on live/dead cells derived from superficial and deep zone of temporomandibular joint (TMJ) condylar cartilage. The cells were treated with and without FFSS stimulation. (**a**) H&E staining of superficial zone and deep zone TMJ cartilage from 3-week-old female rats. Cells from the superficial and deep zone were used for FFSS loading experiments. (**b**,**c**) Typical images of live/dead staining in cultured superficial (**b**) and deep zone cells (**c**) treated with 4 and 24 dyn/cm^2 FFSS. Green, live cells. Red, dead cells. (**d**) Comparison of the percentage of dead cells promoted by the FFSS stimulation. Scale bar, 25 m. n = 3. Results are represented as the mean ± SD. * $P < 0.05$, ** $P < 0.01$, and *** $P < 0.001$ represent significant differences between the indicated groups.

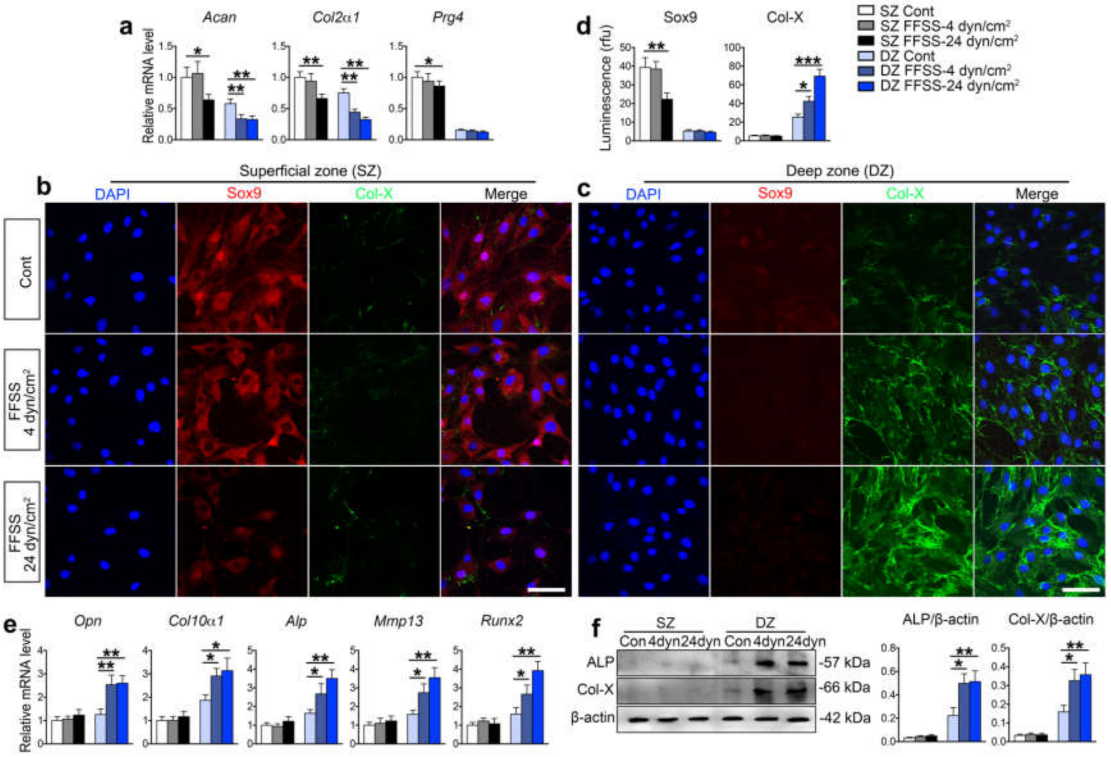

Figure S3. Effects of FFSS on matrix production and cellular terminal differentiation. Primary superficial and deep zone cells were isolated from the mandibular condylar cartilage of rat TMJs and were treated with 4 and 24 dyn/cm² FFSS. (**a**) Comparison of the mRNA expression level of *Acan*, *Col2a1*, and *Prg4*, detected by qPCR analysis, between cells treated with and without FFSS. (**b**–**d**) Immunofluorescence analysis of Sox-9 and Col-X in cultured superficial (**b**) and deep zone cells (**c**) treated with or without FFSS. Representative images (**b** and **c**) and the quantification of immunofluorescence intensity (**d**) are presented. (**e**,**f**) Comparison of the mRNA and protein expression levels in superficial and deep zone cells for terminal differentiation, detected by qPCR analysis and Western blot. Scale bar, 25 m. *n* = 3. Results are represented as the mean ± SD. * $P < 0.05$, ** $P < 0.01$, and *** $P < 0.001$ represent significant differences between the indicated groups.

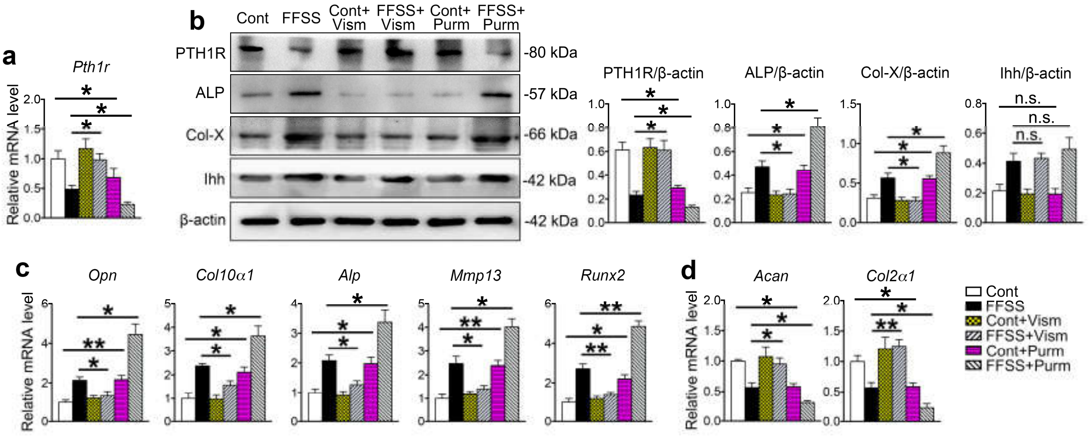

Figure S4. Effects of modified Indian hedgehog (Ihh) signaling on the terminal differentiation of the deep zone cells. (**a**) Comparison of the mRNA expression level of *Pth1r* in deep zone cells from TMJ condylar cartilage between groups treated with 24 dyn/cm^2 FFSS and adding of vismodegib or purmorphamine (1 μM) in the culture medium. (**b**) Western blot analysis for the expression of parathyroid hormone receptor 1(PTH1R), alkaline phosphatase (ALP), type X collagen (Col-X), and Ihh and their quantification. (**c**,**d**) Comparison of mRNA for the markers related to cellular terminal differentiation (**c**) and cartilage matrix (**d**). n = 3. Results are represented as the mean ± SD. * $P < 0.05$, ** $P < 0.01$ represent significant differences between the indicated groups.

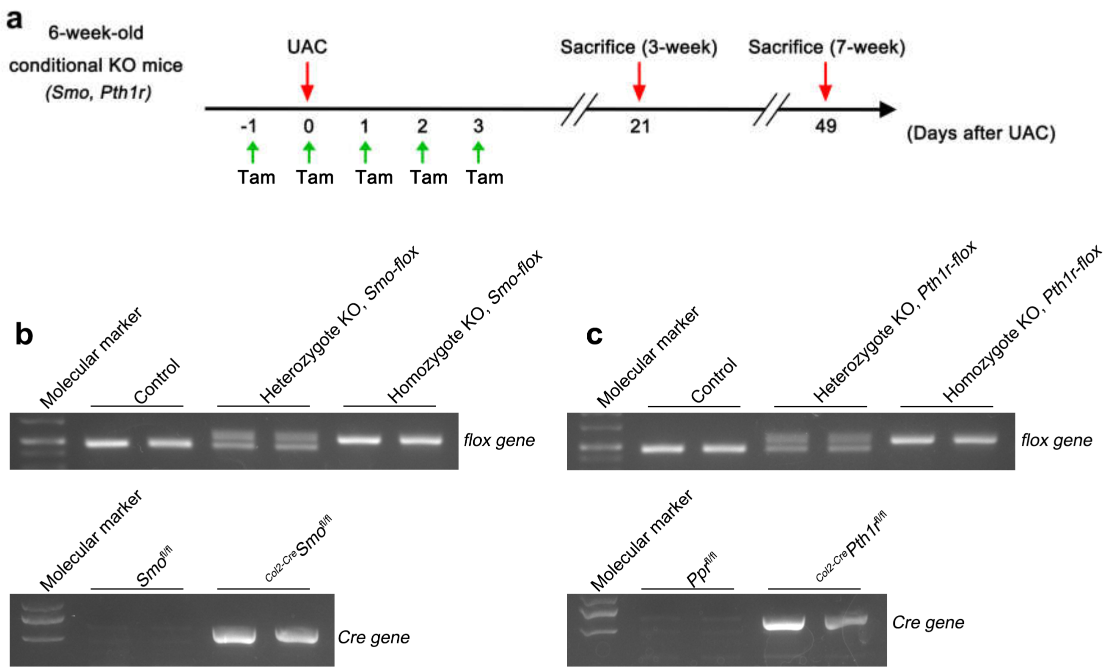

Figure S5. The timeline of whole process of the experiment and genotyping of mice. (**a**) At 6 weeks of age, *Col2-CreER;Smo*$^{fl/fl}$ and *Coll2-Cre;Pth1r*$^{fl/fl}$ mice or double knockout mice were treated with TM (0.1 mg/g of body weight for 5 days). At the second day, UAC was applied. (**b**) Genotyping confirmed the presence of the *Cre* and *flox* transgene in *Col2-CreER;Smo*$^{fl/fl}$ homozygote mice and its absence in wild type mice. (**c**) Genotyping confirmed the presence of the *Cre* and *flox* transgene in *Col2-CreER;Pth1r*$^{fl/fl}$ homozygote mice and its absence in wild type mice. *Tam*, tamoxifen injection.

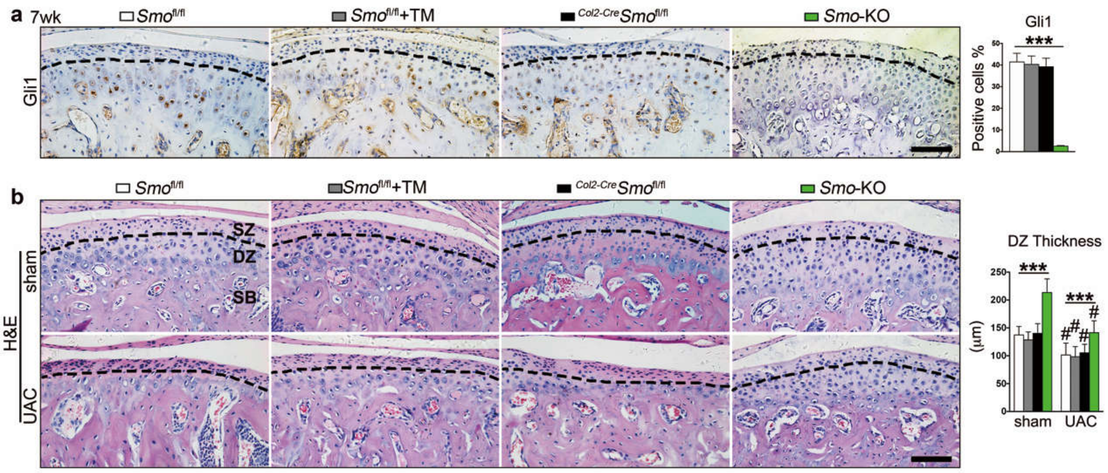

Figure S6. Effect of Ihh signaling inhibition of the TMJ cartilage. (**a**) The Ihh signaling readout of Gli1 was detected by immunohistochemical staining and quantified (right panel). (**b**) Comparison of cartilage thickness, representative images from H&E staining, and the quantitative data between groups. *Smo*$^{fl/fl}$, *Smo*$^{fl/fl}$ + TM, and $^{Col2\text{-}CreER}$*Smo*$^{fl/fl}$: The genetic control mice (see text for details). *Smo*-KO: *Col2-CreER;Smo*$^{fl/fl}$ mice treated with tamoxifen (TM) for deletion of *Smo* gene. UAC treatment was applied from 6-weeks-old and persisted for 7 weeks before the mice being sacrificed. Black dotted lines distinguish the superficial and deep zone chondrocyte. *SZ*, superficial zone. *DZ*, deep zone. *SB*, subchondral bone. Scale bar, 100 m. *n* = 6. Results are represented as the mean ± SD. *** *P* < 0.001 represent significant differences between the indicated groups. # *P* < 0.05 represent significant differences between the sham and UAC group in the four genetic groups.

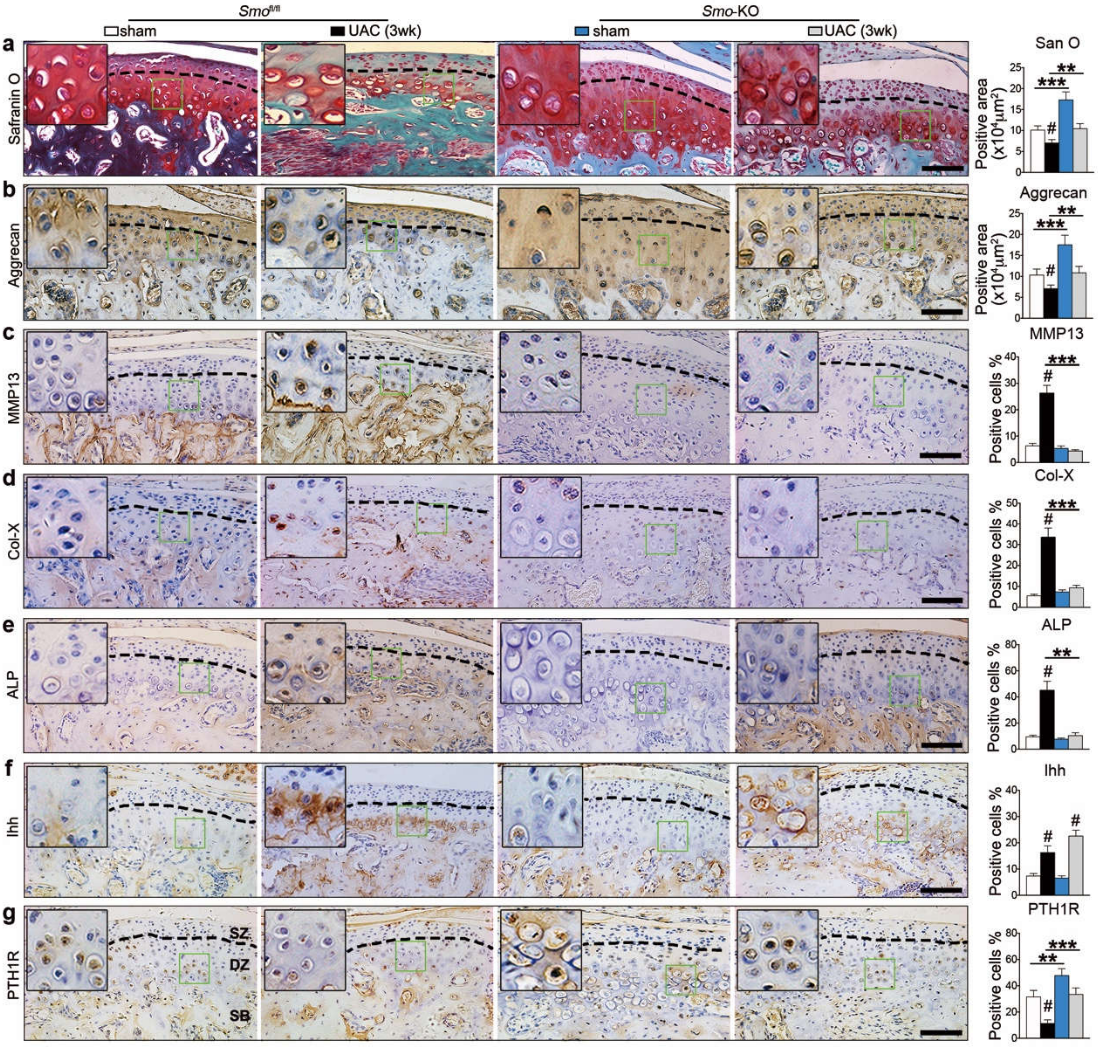

Figure S7. Effect of inhibiting Ihh signaling on cartilage matrix and cellular terminal differentiation in UAC treated mice. Cartilage-specific and tamoxifen-inducible *Smo* gene knockout mice (*Smo*-KO) were treated with 3 weeks of UAC from 6 weeks old. UAC sham operation was delivered in the sham control groups. The *Smo*$^{fl/fl}$ mice were used as the genetic control. (**a**) Safranin O (San O) staining. (**b**) IHC staining of Aggrecan. (**c**–**g**) Immunohistochemical staining of MMP-13, Col-X, ALP, Ihh, and PTH1R. Quantitative data are present in the right panels. The green box regions in the images were magnified at the top left corner. Black dotted lines distinguish the superficial and deep zone chondrocyte. *SZ*, superficial zone. *DZ*, deep zone. *SB*, subchondral bone. Scale bar, 100 μm. $n = 6$. Results are represented as the mean ± SD. ** $P < 0.01$, *** $P < 0.001$ represent significant differences between knockout group and genetic control group. # $P < 0.05$ represent significant differences between the sham and UAC group in the *Smo*$^{fl/fl}$ control group.

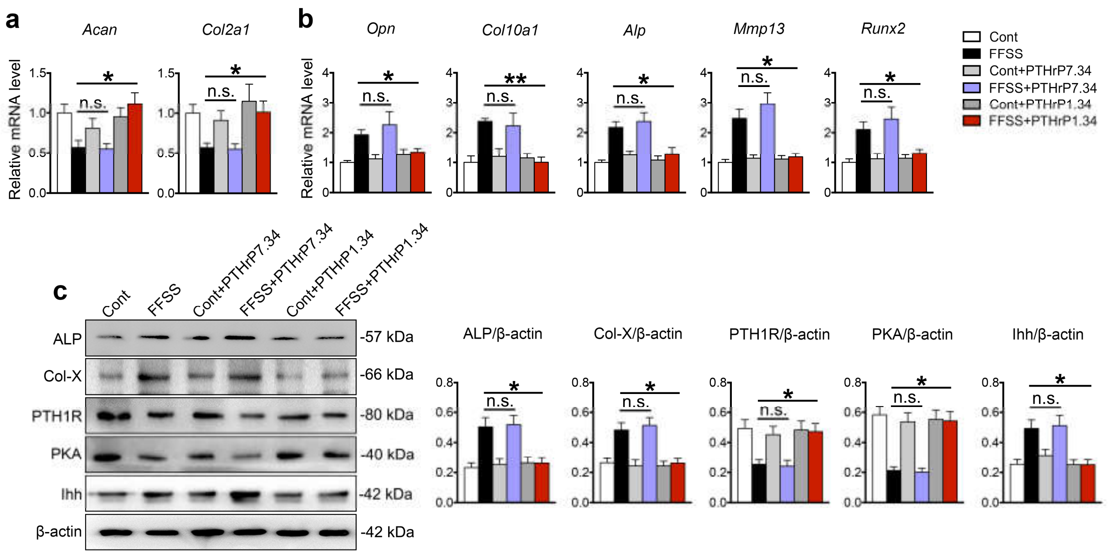

Figure S8. Effect of activation of PTH1R signaling on the terminal differentiation of the deep zone cells treated by FFSS. Comparisons were performed between groups treated with or without PTHrP7.34, the invalid PTHrP legend or PTHrP1.34, the effective PTHrP legend, under the treatment of 24 dyn/cm^2 FFSS. (**a**,**b**) Comparison of the mRNA expression levels for the markers related to cartilage matrix (**a**) and cellular terminal differentiation (**b**). (**c**) Comparison of the protein expression levels for the markers related to ALP, Col-X, PTH1R, PKA, and Ihh. n = 3. Results are represented as the mean ± SD. * $P < 0.05$, ** $P < 0.01$ represent significant differences between the indicated groups.

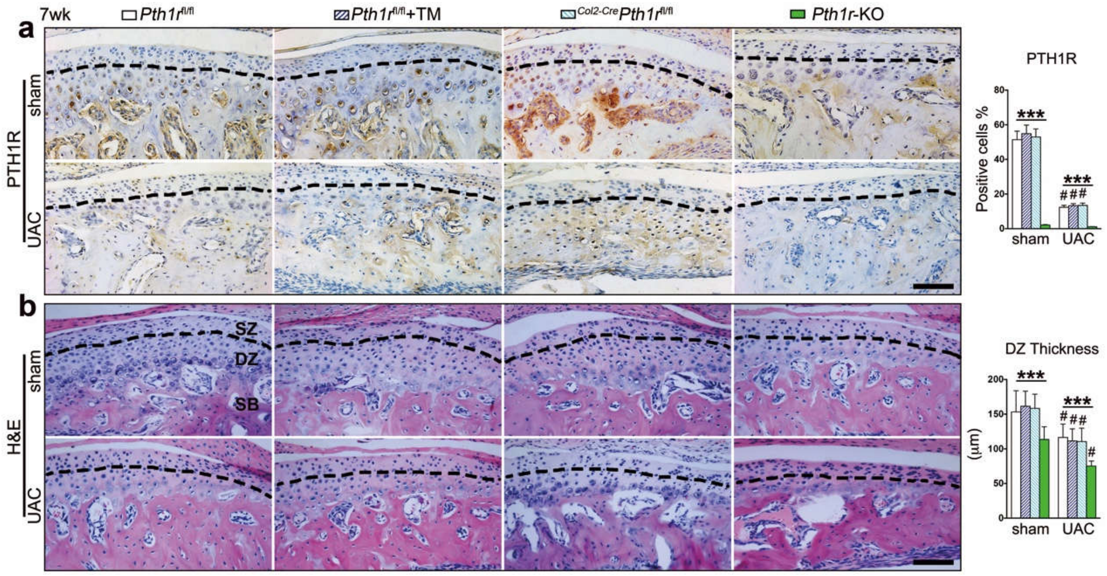

Figure S9. Effect of inhibiting PTH1R signaling on TMJ cartilage of UAC treated mice. Cartilage-specific and tamoxifen-inducible *Pth1r* gene knockout mice (*Pth1r*-KO) were treated with 7 weeks of UAC from 6 weeks old. *Pth1r*$^{fl/fl}$, *Pth1r*$^{fl/fl}$ + TM, and $^{Col2\text{-}CreER}$*Pth1r*$^{fl/fl}$ were used as the genetic controls. (**a**) The expression level of PTH1R detected by immunohistochemical staining. (**b**) H&E staining. Black dotted lines distinguish the superficial and deep zone chondrocyte. *SZ*, superficial zone. *DZ*, deep zone. *SB*, subchondral bone. Scale bar, 100 m. *n* = 6. Results are represented as the mean ± SD. *** $P < 0.001$ represent significant differences between the indicated groups. # $P < 0.05$ represent significant differences between the sham and UAC group in the four genetic groups.

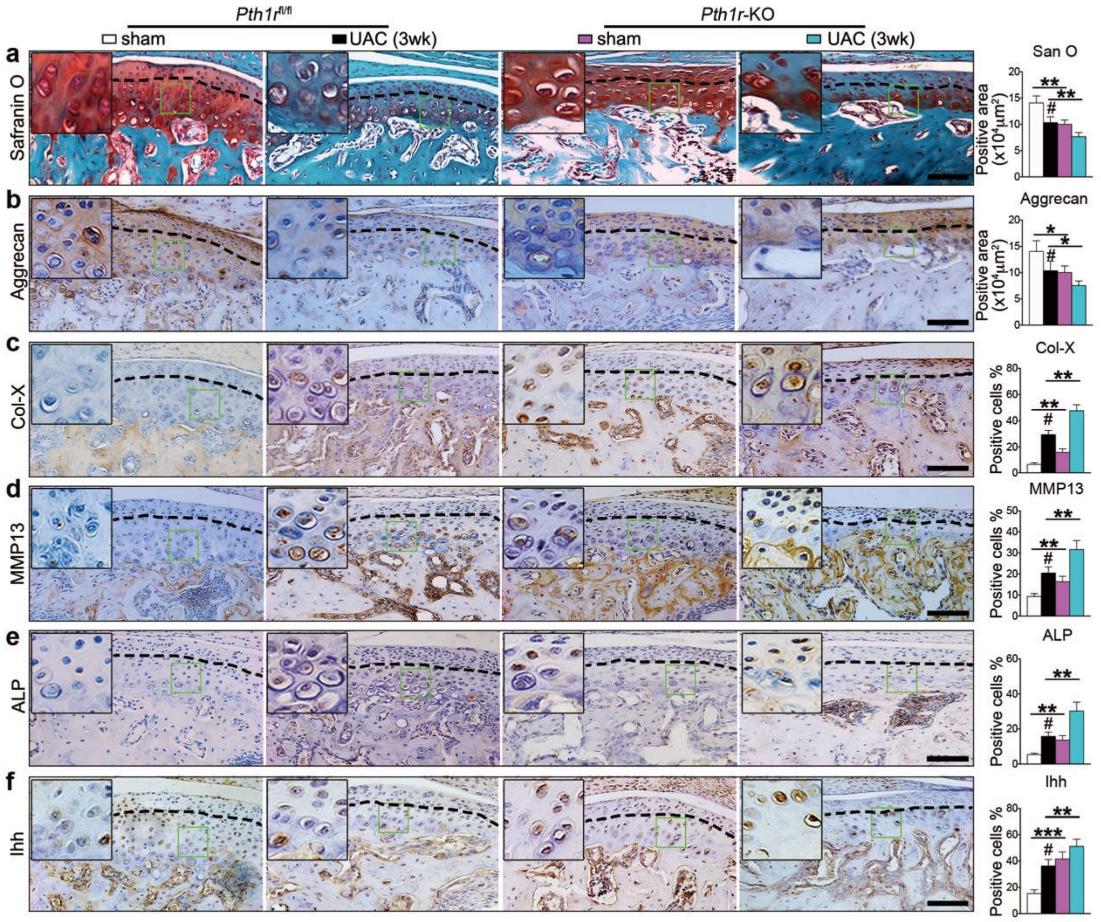

Figure S10. Effect of inhibiting PTH1R signaling on TMJ cartilage matrix in UAC treated mice. The grouping is the same as Figure S9. (**a**) Safranin O staining analysis. (**b**) IHC staining of Aggrecan. (**c**–**f**) Immunohistochemical staining of Col-X, MMP-13, ALP and Ihh. Quantitative data are presented in the right panels. Scale bar, 100 m. The green box regions in the images were magnified at the top left corner. Black dotted lines distinguish the superficial and deep zone chondrocyte. *SZ*, superficial zone. *DZ*, deep zone. *SB*, subchondral bone. Scale bar, 100 m. n = 6. Results are represented as the mean ± SD. * $P < 0.05$, ** $P < 0.01$, *** $P < 0.001$ represent significant differences between knockout group and genetic control group. # $P < 0.05$ represent significant differences between the sham and UAC group in the *Pth1r*$^{fl/fl}$ control group.

Table S1. Primers for genotyping.

Genes	Forward primer	Reverse primer
Smo-flox	ATGGCCGCTGGCCGCCCCGTG	GGCGCTACCGGTGGATGTGG
Pth1r-flox	ATGAGGTCTGAGGTACATGGCTCTGA	CCTGCTGACCTCTCTGAAAGAATGT
Col2-Cre	CGCGGTCTGGCAGTAAAAACTATC	CCCACCGTCAGTACGTGAATATC

Table S2. PCR primers and sequences.

Genes	Forward primer	Reverse primer
Acan	TTCCACCAGTGCGATGCAG	TGGTGTCCCGGATTCCGTA
Col2a1	GAGGGCAACAGCAGGTTCAC	GCCCTATGTCCACACCAAATTC
Prg4	AACAGGGAAGATAGTGGC	CGTAGTAATCATAGCCGTCA
Alp	CACGTTGACTGTGGTTACTGCTGA	CCTTGTAACCAGGCCCGTTG
Col10a1	GTTCTTGACCCTGGTTCA	CTGAGGGACCTGGGTGT
Mmp13	TCCCTGGAATTGGCAACAAAG	GCATGACTCTCACAATGCGATTAC
Runx2	CATGGCCGGGAATGATGAG	TGTGAAGACCGTTATGGTCAAAGTG
Osx	CACCCATTGCCAGTAATCTTCGT	GGACTGGAGCCATAGTGAGCTTC
Ihh	CTTCAGCGATGTGCTCATTT	ATGATTGTCCGCAATGAAGA
Smo	ACAGTTCAGGCCAATGTGAA	AAGGCTGCGATGTAACTGTG
Pthrp	ATGACAAGGGCAAGTCCATC	CGTCTCCACCTTGTTGGTTT
Pth1r	AGGTGGTTCCAGGGCACAA	CAACTCTTCCTCTGTGAGGC
Gapdh	GGCACAGTCAAGGCTGAGAATG	ATGGTGGTGAAGACGCCAGTA
